# Treatment of persistent trophoblastic disease later than 6 months after diagnosis of molar pregnancy

**DOI:** 10.1054/bjoc.1999.1124

**Published:** 2000-03-21

**Authors:** A M Gillespie, S Kumar, B W Hancock

**Affiliations:** Trophoblastic Disease Screening and Treatment Centre, Yorkshire Cancer Research Department of Clinical Oncology, We ston Park Hospital, Whitham Road, Sheffield, S10 2SJ, UK

**Keywords:** trophoblastic disease, late treatment, molar pregnancy

## Abstract

Of 4257 patients with gestational trophoblastic disease (GTD) registered between 1986 and 1996 with the Trophoblastic Screening and Treatment Centre, Sheffield, 231 women required chemotherapy; 28 were treated 24 weeks or more after the initial evacuation of products of conception. In 18 patients late treatment was a result of a predetermined watch and wait policy on the part of the Centre; these patients formed the study group. Patients were identified from the Centre's computer database. The time interval from first evacuation (diagnosis) to start of chemotherapy was calculated for each patient. Hospital records were reviewed when the interval of observation was 24 weeks or greater to determine patient characteristics, treatment and outcome. Eighteen women were treated ‘late’ (according to Centre policy), with a median age of 30 years (range 21–57 years). The interval from diagnosis to treatment ranged from 24 to, in one case, 56 weeks (median 33 weeks). Fourteen of 18 women had complete moles, 3/18 had partial moles and one had unclassified disease. All women had low-risk disease and were treated with single-agent methotrexate; 17 were cured with this regimen, one also required salvage chemotherapy. In conclusion, where a successful surveillance programme is in operation for GTD, a wait and watch policy can be adopted without compromising patients whose definitive treatment is commenced more than 6 months after the initial diagnosis. © 2000 Cancer Research Campaign


British Journal of Cancer (2000) **82**(8), 1393–1395
© 2000 Cancer Research Campaign
DOI: 10.1054/ bjoc.1999.1124, available online at http://www.idealibrary.com on IDEAL®

### Treatment of persistent trophoblastic disease later than 6 months after diagnosis of molar pregnancy

**AM Gillespie, S Kumar and BW Hancock**

Trophoblastic Disease Screening and Treatment Centre, Yorkshire Cancer Research Department of Clinical Oncology, Weston Park Hospital, Whitham Road, Sheffield S10 2SJ, UK

**Summary** Of 4257 patients with gestational trophoblastic disease (GTD) registered between 1986 and 1996 with the Trophoblastic Screening and Treatment Centre, Sheffield, 231 women required chemotherapy; 28 were treated 24 weeks or more after the initial evacuation of products of conception. In 18 patients late treatment was a result of a predetermined watch and wait policy on the part of the Centre; these patients formed the study group. Patients were identified from the Centre's computer database. The time interval from first evacuation (diagnosis) to start of chemotherapy was calculated for each patient. Hospital records were reviewed when the interval of observation was 24 weeks or greater to determine patient characteristics, treatment and outcome. Eighteen women were treated 'late' (according to Centre policy), with a median age of 30 years (range 21–57 years). The interval from diagnosis to treatment ranged from 24 to, in one case, 56 weeks (median 33 weeks). Fourteen of 18 women had complete moles, 3/18 had partial moles and one had unclassified disease. All women had low-risk disease and were treated with single-agent methotrexate; 17 were cured with this regimen, one also required salvage chemotherapy. In conclusion, where a successful surveillance programme is in operation for GTD, a wait and watch policy can be adopted without compromising patients whose definitive treatment is commenced more than 6 months after the initial diagnosis. © 2000 Cancer Research Campaign

**Keywords:** trophoblastic disease; late treatment; molar pregnancy

Gestational trophoblastic disease (GTD) is an aberration of the trophoblast consisting of a wide spectrum of diseases ranging from benign hydatidiform mole to highly malignant choriocarcinoma and placental site trophoblastic tumour. The UK Trophoblastic Disease Registration Scheme was initiated in 1973 by the Royal College of Obstetricians and Gynaecologists. There are three supra-regional centres in the UK: in London (Charing Cross Hospital), in Sheffield (Weston Park Hospital), and in Dundee (Ninewells Hospital). The Sheffield team treats by chemotherapy approximately 5% of all patients registered with this centre – a lower proportion than in other treatment centres worldwide. This figure was achieved by adopting stringent treatment criteria and definitions of persistent trophoblastic disease (PTD).

It has been suggested that such a treatment policy can result in delayed initiation of chemotherapy in a small subgroup of patients and that patients who present late may have a poorer prognosis. This study evaluates the characteristics and outcome of patients whose chemotherapy was initiated more than 24 weeks after initial diagnosis as part of a predetermined policy on the part of the Centre.

### MATERIALS AND METHODS

Patients with GTD are registered after diagnosis at the Trophoblastic Disease Screening and Treatment Centre. They are then placed on a surveillance programme which mainly consists of serial measurements of the tumour marker, human chorionic gonadotrophin (hCG). At our Centre we generally recommend that patients have a repeat evacuation when the tumour marker remains elevated or fails to fall in an optimal manner. In addition, the referring gynaecologist may perform a repeat evacuation if the patient has persistent symptoms.

Patients who have evidence of PTD as determined by the criteria for treatment (Table 1) are admitted for assessment and chemotherapy. The patient is assigned to a risk group (Sheridan et al, 1993) based on the Charing Cross Prognostic Scoring System (Bagshawe, 1976). Patients with low risk (score ≤ 7) receive low-dose methotrexate as the first-line chemotherapy (methotrexate 50 mg intramuscular (i.m.) on alternate days with four doses per cycle, folinic acid 7.5 mg orally 24 h after each injection and 7-day rest periods between cycles). Those who have an inadequate tumour response to first-line therapy are transferred to the EA salvage regimen (etoposide 100 mg $m^{-2}$ intravenously (i.v.) and actinomycin D 0.5 mg i.v. daily for 3 days with rest period of 7 days between each cycle). Patients with high-risk disease (risk score > 7) are treated with the MAE regimen as first-line chemotherapy (methotrexate 100 mg $m^{-2}$ i.v. over an hour followed by a 12 h infusion of 200 mg $m^{-2}$ is added to and alternates with the EA regimen).

The computer database at the Centre was searched to identify patients treated for PTD between 1986 and 1996. The time interval from diagnosis to start of chemotherapy was calculated for each patient. Hospital records were reviewed when the interval was 24 weeks or greater to determine patient characteristics, treatment and outcome.

*Received 30 July 1999*
*Revised 13 November 1999*
*Accepted 8 December 1999*

*Correspondence to:* AM Gillespie

**Table 1** Criteria for chemotherapy in gestational trophoblastic disease at the Sheffield Trophoblastic Screening Centre

- hCG levels > 20 000 IU/l after 2nd or 3rd evacuation
- Static or rising hCG levels after 2nd or 3rd evacuation
- Persistent uterine haemorrhage with elevated hCG level
- Persistent hCG elevation, 6 months after evacuation
- Pulmonary metastasis with static or rising hCG levels
- Metastasis in liver, brain or gastrointestinal tract
- Histological diagnosis of true choriocarcinoma

### RESULTS

A total of 4257 women with gestational trophoblastic disease were registered in Sheffield between 1986 and 1996. In total, 231 of these women required chemotherapy for PTD or choriocarcinoma. Twenty-eight patients were treated 'late', with chemotherapy commencing 24 weeks or more following initial diagnosis. In five cases this was due to late recurrence of GTD; in five cases there was delayed referral to the Centre or defaulting follow-up on the part of the patient. Thus, 18 patients fulfilled the criteria for our watch and wait policy (i.e. to treat persistent βhCG elevation 6 months post-uterine evacuation).

The median patient age of these 18 patients was 30 years (range 21–57 years). Sixteen patients had suction curettage as initial mode of evacuation (one was medically induced) and the other two patients spontaneously miscarried their molar pregnancies. On histological analysis, 14 had complete hydatidiform moles, three had partial moles, one had an unclassified mole.

Consistent with previous Unit policy most patients had repeat uterine evacuations prior to initiation of chemotherapy. All repeat evacuations were performed by dilatation and curettage. Fourteen of 18 patients had one repeat evacuation and a further 4/18 patients had two repeat procedures. Repeat evacuation(s) was performed in seven cases for continued vaginal bleeding and in 11 for a persistent or rising hCG levels.

βhCG levels at registration (ranging from 2 to 6 weeks post-diagnosis) were 66–72 000 (mean 11 300) iu/l. At commencement of chemotherapy the range was 63–3300 (mean 950) iu/l.

Chemotherapy was commenced at a median of 33 weeks (range 24–56 weeks) after the initial evacuation. Charing Cross Hospital prognostic scores ranged from 3 to 6, with a median of 4. All patients had low-risk disease (risk score of ≤ 7); all were started on single-agent methotrexate, one required salvage therapy with the EA regimen. All 18 patients are in complete remission. All patients tolerated chemotherapy well and with minimal clinically significant side-effects.

During the period of the study, 17 patients were observed for 24 weeks or longer (range 24–44, mean 30) and did not require chemotherapy because the βhCG level fell spontaneously to normal. In this group repeat evacuations were done in 10/17 patients (in one case two re-evacuations were performed) – in six cases for elevated βhCG levels, in four for persistent vaginal bleeding. βhCG levels at registration were 100–20 000 (mean 5700) iu/l.

### DISCUSSION

At the Sheffield Trophoblastic Disease Centre we are able to successfully adopt stringent treatment criteria for PTD for three reasons:

1. trophoblastic tumours are generally exquisitely chemosensitive
2. hCG measurements accurately reflect the tumour burden
3. excellent patient compliance with an inclusive national surveillance scheme.

The chemotherapy policy (Table 1), including the use of repeat uterine evacuation – which leads to disease resolution in up to three-quarters of patients in whom this is performed, has enabled us to expose only 5% of patients registered in our centre to cytotoxic chemotherapy, whilst reporting very high overall cure rates (Sheridan et al, 1993; Hancock, 1997). In the USA, the mobility of the population and medico-legal factors result in less stringent criteria being used – women with βhCG not regressing to normal in 4–6 weeks receive chemotherapy. This results in 20–30% of all patients receiving cytotoxic agents (Goldstein and Berkowitz, 1982; Lurain et al, 1983). We conducted this study to assess whether adopting a stringent policy resulted in increased morbidity and mortality amongst those who fulfilled our treatment criteria 6 months or more after initial diagnosis.

Overall, 20% of low-risk disease patients initially treated with single-agent methotrexate chemotherapy develop resistance, but in our experience all such patients can be cured with salvage chemotherapy (Sheridan et al, 1993). In this series all patients had low-risk disease and all achieved complete remission. One patient who developed drug resistance was cured with combination chemotherapy (etoposide/dactinomycin). In the period between 1986 and 1996, the Centre had five deaths out of 231 patients patients treated with chemotherapy for PTD. It would seem therefore that mortality is not increased in the 'late' treated group.

If mortality is not affected, is there any evidence that this patient group suffers from increased morbidity? All patients were assessed as low risk; in addition there was no evidence that more chemotherapy was required to induce and maintain remission. No patient had serious drug-associated toxicity. It is, therefore, concluded that there was no increased morbidity in the late treated group.

An observation of note in this patient group is the number of patients with partial hydatidiform moles. It is known that partial moles rarely develop into PTD (Berkowitz and Goldstein, 1997). In this series of 18 cases, three histologically confirmed partial moles were documented. These three cases represent one-third of all partial moles requiring chemotherapy at our Centre between during the period of study. It could be argued that our surveillance programme allows the monitoring of the natural history of a partial mole; that these tumours have a more indolent course than other forms of GTD and that patients with such tumours can safely be monitored for prolonged periods rather than rapidly initiating chemotherapy.

The more stringent the treatment criteria adopted for chemotherapy of PTD the smaller the proportion of patients who will require treatment. This is important as it has been shown that, whilst chemotherapy regimens are highly efficacious, even the least toxic regimens used can be associated with significant side-effects (Gillespie et al, 1997; Sharma et al, 1999) and the more rigorous regimens can be associated with long-term sequelae (Rustin et al, 1987, 1996). A stringent treatment policy does mean that definitive chemotherapy will be delayed in some patients. Our current treatment policy dictates that chemotherapy is required for those patients with persistently elevated βhCG 6 months after initial diagnosis. However, even in this group we are sometimes justified in adopting a 'watch and wait' strategy as during the time

© 2000 Cancer Research Campaign period of this study 17 patients were observed closely for 24 weeks or longer and did not require chemotherapy, their persistent trophoblastic disease eventually resolving spontaneously. This explains the range of intervals to treatment in those patients requiring chemotherapy.

The study indicates that there is no increased morbidity or mortality in patients monitored closely for 6 months or more before initiation of chemotherapy. We conclude that such a strategy can be safely adopted in the management of PTD where effective surveillance procedures are available.

### REFERENCES

Bagshawe KD (1976) Risk and prognostic factors in trophoblastic neoplasia. *Cancer* **38**: 1373–1385

Berkowitz RS and Goldstein DP (1997) Presentation and management of molar pregnancy. In: *Gestational Trophoblastic Disease*, Hancock BW, Newland ES and Berkowitz RS (eds), pp. 127–142. Chapman & Hall: London

Gillespie AM, Lorigan PC, Radstone CR, Waterhouse JC, Coleman RE and Hancock BW (1997) Pulmonary function in patients with trophoblastic disease treated with low-dose methotrexate. *Br J Cancer* **76**: 1382–1386

Goldstein DP and Berkowitz RS (1982) The diagnosis and management of molar pregnancy. In: *Gestational Trophoblastic Neoplasms*: *Clinical Principles of Diagnosis and Management*, pp. 143–175. WB Saunders: Philadelphia

Hancock BW (1997) Differences in management and treatment: a critical appraisal. In: *Gestational Trophoblastic Disease*, Hancock BW, Newlands ES and Berkowitz RS (eds), pp. 213–218. Chapman & Hall: London

Lurain JR, Brewer JI, Torok EE and Halpern B (1983) Natural history of hydatidiform mole after primary evacuation. *Am J Obstet Gynecol* **145**: 591–595

Rustin GJS, Pektasides D, Bagshawe KD, Newlands ES and Begent RHJ (1987) Fertility after chemotherapy for male and female germ cell tumours. *Inst J Androl* **10**: 389–392

Rustin GJS, Newlands ES, Lutz JM, Holden L, Bagshawe KD, Hiscox JG, Foskett M, Fuller S and Short D (1996) Combination but not single-agent methotrexate chemotherapy for gestational trophoblastic tumours increases the incidence of second tumours. *J Clin Oncol* **14**: 2769–2773

Sharma S, Jagdev S, Coleman RE, Hancock BW and Lorigan PC (1999) Serosal complications of single agent low dose methotrexate used in gestational trophoblastic diseases: first reported case of methotrexate induced peritonitis. *Br J Cancer* **81**: 1037–1041

Sheridan E, Hancock BW, Smith SC, Dorreen MS, Neal FE, Pennington GW and Millar DR (1993) Gestational trophoblastic disease. Experience of the Sheffield (United Kingdom) supraregional screening and treatment service. *Int J Oncol* **3**: 149–155

© 2000 Cancer Research Campaign
